# Non-Steroidal Anti-Inflammatory Drugs, Variation in Inflammatory Genes, and Aggressive Prostate Cancer

**DOI:** 10.3390/ph3103127

**Published:** 2010-10-08

**Authors:** Adam C. Reese, Jill Hardin, Iona Cheng, Graham Casey, John S. Witte

**Affiliations:** 1 Department of Urology, University of California at San Francisco, 1450 3^rd^ Street, San Francisco, CA 94158, USA; E-Mail: areese@urology.ucsf.edu (A.C.R.); 2 Epidemiology and Biostatistics and Institute for Human Genetics, University of California at San Francisco, 1450 3^rd^ Street, San Francisco, CA 94158, USA; E-Mail: hardinj@humgen.ucsf.edu (J.H.); 3 Epidemiology Program, Cancer Research Center of Hawai`i, University of Hawai`i, Honolulu, HI 96813, USA; 4 Department of Preventive Medicine, University of Southern California, Los Angeles, CA 90033, USA

**Keywords:** prostatic neoplasms, non-steroidal anti-inflammatory agents, aspirin, genetic variation, single nucleotide polymorphism

## Abstract

Increasing evidence suggests that prostatic inflammation plays a key role in the development of prostate cancer. It remains controversial whether non-steroidal anti-inflammatory drugs (NSAIDs) reduce the risk of prostate cancer. Here, we investigate how a previously reported inverse association between NSAID use and the risk of aggressive prostate cancer is modulated by variants in several inflammatory genes. We found that NSAIDs may have differential effects on prostate cancer development, depending on one’s genetic makeup. Further study of these inflammatory pathways may clarify the mechanisms through which NSAIDs impact prostate cancer risk.

## 1. Introduction

Chronic inflammation has been implicated as a causative factor in a wide range of malignancies, including lung, colorectal, pancreatic, bladder, hepatocellular, and prostatic carcinomas [1]. Should inflammation play a role in carcinogenesis, it follows naturally that anti-inflammatory interventions may help limit cancer development and progression. Thus, much research in recent years has focused on the potential chemoprotective effects of non-steroidal anti-inflammatory drugs (NSAIDs) [2]. This group of medications, of which ibuprofen and aspirin (ASA) are the most prominent, act as inhibitors of cyclooxygenase (COX), a key enzyme in the pro-inflammatory pathway. NSAIDs are widely used, both acutely and chronically, for their analgesic, anti-pyretic, and anti-inflammatory effects. Furthermore, the potential cardioprotective effect of aspirin has markedly increased the number of chronic NSAID users in recent years [3,4]. 

The strongest data supporting an anti-cancer effect of NSAIDs has been reported in the gastroenterology literature, where both ASA and non-ASA NSAIDs have been shown to decrease the risk of colorectal cancer [5]. Less convincing observational evidence has reported a chemoprotective effect of NSAIDs in esophageal, gastric, lung, and breast cancer [2]. Whether NSAID use alters one’s risk of prostate cancer—the most common non-cutaneous malignancy diagnosed in men—remains unclear. Although experimental work shows that NSAIDs may inhibit prostate cancer cell growth [6,7,8], increase apoptosis [6,8,9], and decrease angiogenesis [6], these promising findings have not been consistently replicated in epidemiological studies. Many studies have reported a decreased risk of prostate cancer in NSAID users [10,11,12,13,14,15,16,17,18,19,20,21], but others have reported no association [22,23,24,25,26,27,28,29], and some have found NSAID use to actually increase risk of prostate cancer [30,31]. Thus, the impact of NSAIDs on prostate carcinogenesis remains unclear. 

Furthermore, the effects of NSAIDs on the development of advanced or aggressive disease is even less clear. Understanding risk factors for more aggressive disease is particularly important given the widely heterogeneous behavior of prostate cancer. Whereas high grade, advanced disease is frequently rapidly progressive and ultimately fatal, low grade, localized tumors often pose little risk to the patient in terms of symptoms or cancer-related death [32]. 

Finally, the mechanisms through which NSAIDs may affect prostate cancer development are not fully understood. Although most investigators have presumed NSAIDs act through COX-2 inhibition [33], others have proposed that the anti-tumor effects of NSAIDs are mediated through COX-2-independent mechanisms [7,34].

We have previously reported a decreased risk of advanced/aggressive prostate cancer in NSAID users relative to non-users [15]. Additionally, the effect of NSAID use on prostate cancer susceptibility appeared to be modified by genetic variants in two inflammatory genes, *COX-2* and lymphotoxin-alpha (*LTA*) [10,15]. Whereas NSAID use was found to lower the risk of prostate cancer in carriers of a particular sequence variant of *COX-2* and *LTA*, there was no association between NSAIDs and disease risk in men of alternate genotypes [10,15]. These findings suggest that *COX-2* and *LTA* may be key players in the mechanism through which NSAIDs alter prostate cancer risk. 

In the current study, we have expanded our investigation of NSAIDs to consider additional genes involved with inflammation, in hopes of further identifying variants that modulate the association between NSAIDs and development of aggressive prostate cancer. These findings may identify candidate proteins with which NSAIDs interact, potentially altering prostate cancer causative pathways. 

## 2. Experimental Section

### 2.1. Study Subjects

The study population consisted of 1,034 men, recruited from the major medical institutions in Cleveland, Ohio (The Cleveland Clinic, University Hospital of Cleveland, and their affiliates). This population included 499 cases with newly-diagnosed, advanced/ aggressive prostate cancer and 535 controls frequency matched by age, race, and treating institution, recruited between 2001 and 2004. In an attempt to study only those tumors thought to be clinically relevant, cases were restricted to those men with either aggressive or advanced disease, defined by clinical stage ≥T2c, PSA at diagnosis >10 ng/mL, or biopsy Gleason score ≥7. Controls were recruited from a group of men who underwent standard medical examinations at the participating institutions. Controls had no history of prostate or other non-cutaneous malignancy. All controls were screened for prostate cancer with serum PSA, and those with PSA > 4ng/mL were encouraged to undergo urological evaluation. Of 50 controls with an elevated PSA, two patients were subsequently found to have prostate cancer. These two patients met our definition for advanced/ aggressive disease and were thus reclassified as cases. Detailed information and descriptive characteristics for this case-control study has been reported previously [15].

### 2.2. NSAID Use

Study participants were asked about the amount and duration of previous aspirin- and ibuprofen-containing drug use (prior to diagnosis for cases and prior to enrollment for controls) during a personal interview. We defined any NSAID consumption as use of either aspirin or ibuprofen at least twice a week for more than one month, and never use as no reported use for both medications. We then calculated “NSAID pill-years” in order to determine the dose and duration of NSAID use. NSAID pill-years was calculated as the product of the number of pills taken per day and the years of drug use. The summation of pill-years for aspirin and ibuprofen was calculated to determine the total dose/duration of NSAID use.

### 2.3. SNP Selection

In order to determine whether genetic variation modulated the effect of NSAIDs on prostate cancer risk, we focused on SNPs in candidate genes potentially involved with innate immunity and inflammation (e.g., cyclooxygenase-2 (*COX-2*), toll-like receptor 4 (*TLR4*), transforming growth factor beta-1 (*TGFβ1*), interleukin-12 subunit beta (*IL-12β*), and macrophage scavenger receptor 1 (*MSR1*)). We chose to focus on such candidate genes because the anti-inflammatory effects of NSAIDs would logically interact with their gene products– proteins that serve as key players in several inflammatory pathways. We evaluated interactions for SNPs exhibiting an independent association with risk of prostate cancer in our data (*i.e.*, irrespective of NSAID use). In particular, we first identified 311 SNPs, including tag SNPs (r^2^ > 0.80) with the unmeasured SNP, potentially functional SNPs, as well as those previously associated with prostate cancer that spanned 43 candidate genes involved in innate immunity and inflammation. We then investigated the independent associations between theses SNPs and prostate cancer risk; 52 SNPs in 22 of the genes were associated with the risk of aggressive disease irrespective of NSAID use (p-values ≤ 0.05) and so were studied here for their potential interaction with NSAIDs (not shown; details on all candidate genes and SNPs available on request). 

### 2.4. Genotyping

Genotyping was performed using the TaqMan allelic discrimination assay or the Illumina GoldenGate^®^ assay. All assays were performed by individuals blinded to case-control status. For quality control, 2% replicate samples were included. The concordance rate for replicate samples was >99%. The average genotyping success rate was >98%. All SNPs were in Hardy-Weinberg equilibrium (at *P* > 0.01 level).

### 2.5. Statistical Analysis

Unconditional logistic regression was used to calculate the odds of advanced/aggressive prostate cancer in NSAID users relative to non-users. Patients were then stratified into quintiles based on dose-duration of NSAID use in controls, and the odds of disease was calculated for each quintile, relative to the lowest quintile of NSAID use. To test for confounding, separate models were run controlling for BMI and family history of prostate cancer. Using a threshold of the change in effect estimates of 10% or more, there was no evidence that BMI or family history of prostate cancer acted as confounders. 

Men were then stratified by genotype of the 52 SNPs evaluated here. Unconditional logistic regression was used to determine the odds of disease in NSAID-users relative to non-users in patients of each genotype. We also tested the association for dose/duration of NSAIDs (categorized into quintiles based on the distribution in controls). To examine interactions between genotypes and NSAID use, we dichotomized the latter into use and no use and assessed the effect of NSAID use by genotypes in log additive or dominant models. To test for statistical interaction, the model contained separate terms for the main effect of genotype, the main effect of NSAID use, and an interaction term for the cross product of genotype and NSAID use. To account for multiple comparisons, permutation testing was performed 100 times for each of the six NSAID x SNP interactions shown in Table 5 and permutation adjusted p values are presented. 

All odds ratio estimates were adjusted for age, race, and treating institution. All reported p-values are two sided. For the sake of brevity we only present results exhibiting nominally significant interactions; all results are available upon request.

## 3. Results and Discussion

The study was comprised of 1,034 study subjects (prostate cancer cases = 499, controls = 535). Patient demographics and serum PSA of cases and controls are shown in table 1. Most patients were Caucasian (81.0%) and over 60 years of age (73.3%). The distribution of serum PSA in the cases reflects our selection of patients meeting PSA criteria for advanced/aggressive disease (PSA > 10 ng/mL). 

**Table 1:** Characteristics of study population (advanced /aggressive prostate cancer cases and controls).

	**Cases (n = 499)**		**Controls (n = 535)**	
Age (years), mean (SD)	65.9	(8.4)	65.9	(8.5)
Race, N (%)				
Caucasian	407	(81.6)	431	(80.6)
African-American	92	(18.4)	104	(19.4)
Education, N (%)				
<12 years	49	(9.8)	51	(9.5)
12 years or high school	112	(22.4)	78	(14.6)
Some college	105	(21.0)	95	(17.8)
≥ College graduate	233	(46.7)	311	(58.1)
Family history of prostate cancer^1,2^, N (%)				
Negative	384	(77.0)	475	(89.0)
Positive	115	(23.1)	59	(11.0)
Smoking, N (%)				
Never	200	(40.1)	217	(40.6)
Former	58	(11.6)	48	(9.0)
Current	241	(48.3)	270	(50.5)
Body mass index (kg/m^2^) mean (SD)	26.3	(3.9)	26.4	(3.7)
Serum PSA value^3^ (ng/ml) , N (%)				
≤4.0	26	(5.2)	493	(92.2)
>4.0 & <10.0	250	(50.1)	35	(6.5)
≥10.0	223	(44.7)	6	(1.1)
Clinical stage^2^, N (%)				
T1	309	(64.6)		
T2a & Tb	128	(26.8)		
T2c	15	(3.1)		
T3 & T4	26	(5.4)		
Total Gleason Grade, N (%)				
<7	75	(15.0)		
7, 3+4	218	(43.7)		
≥7, 4+3	206	(41.3)		

^1^ Positive family history of prostate cancer defined as prostate cancer in a first degree relative. ^2^ Percentages do not add to 100 due to missing values. ^3^ PSA missing for one control subject.

Table 2 shows the demographics and disease characteristics of cases, comparing NSAID-users to non-users. Compared to those patients who did not use NSAIDs, NSAID users were more often Caucasian, were better educated, and were heavier. Although the distribution of clinical stage and Gleason score was relatively similar between the two groups, there was a non-significant trend towards lower serum PSA in NSAID users. These findings suggest that NSAIDs may act to suppress serum PSA, a phenomenon that has been previously reported by several different groups [35,36,37,38]. The decision to perform prostate biopsy is typically triggered by PSA elevation above a certain threshold. Thus, a PSA-suppressing effect of NSAIDs could potentially delay biopsy and subsequent prostate cancer diagnosis in NSAID users. As the clinical ramifications of delayed diagnosis are significant, the potential for PSA suppression by NSAIDs certainly deserves further investigation.

**Table 2:** Characteristics of Aggressive Prostate Cancer Cases (NSAID users compared to non-users).

	**NSAID Use Among Cases**					**NSAID Use Among Controls**				
	Yes (n = 272)		No (n = 227)		P-value^3^	Yes (n = 340)		No (n = 195)		P-value^3^
Age (years), mean (SD)	66.6	(8.1)	66.3	(8.6)	0.07	67.2	(8.1)	66.4	(9.2)	
Race, N %					<0.01					<0.01
Caucasian	241	(88.6)	166	(73.1)		290	(85.3)	141	(72.3)	
African-American	31	(11.4)	61	(26.9)		50	(14.7)	54	(27.7)	
Education, N (%)					<0.01					0.07
<12 years	26	(9.6)	23	(10.1)		24	(7.1)	27	(13.9)	
12 years or high school	44	(16.2)	68	(30.0)		53	(15.6)	25	(12.8)	
Some college	54	(19.9)	51	(22.5)		60	(17.7)	35	(18.0)	
≥College graduate	148	(54.4)	85	(37.4)		203	(59.7)	108	(55.4)	
Smoking, N (%)					0.08					0.96
Never	109	(40.1)	91	(40.1)		139	(40.1)	78	(40.0)	
Former	24	(8.8)	34	(15.0)		31	(9.1)	17	(8.7)	
Current	139	(51.1)	102	(44.9)		170	(50.0)	100	(51.3)	
Family history of prostate cancer^1, 2^, N (%)					0.32					0.32
Negative	214	(78.7)	170	(74.9)		305	(89.7)	170	(87.2)	
Positive	58	(21.3)	57	(25.1)		35	(10.3)	24	(12.3)	
Body mass index (kg/m^2^), mean (SD)	27.1	(4.1)	25.8	(3.3)	0.02	26.9	(3.7)	26.8	(3.8)	
Serum PSA value (ng/ml) ^2^, N (%)					0.11					0.51
≤4.0	16	(5.9)	10	(4.4)		314	(92.4)	179	(91.8)	
>4.0 & <10.0	146	(53.7)	104	(45.8)		23	(6.8)	12	(6.2)	
≥10.0	110	(40.4)	113	(49.8)		3	(0.9)	3	(1.5)	
Clinical stage^2^, N (%)					0.30					
T1	167	(61.4)	142	(62.6)						
T2a & Tb	76	(27.9)	52	(22.9)						
T2c	6	(2.2)	9	(4.0)						
T3 & T4	15	(5.5)	11	(4.9)						
Total Gleason Grade, N (%)					0.68					
<7	39	(14.3)	36	(15.9)						
7, 3+4	116	(42.7)	102	(44.9)						
≥7, 4+3	117	(43.0)	89	(39.2)						

^1^Positive family history of prostate cancer defined as prostate cancer in a first degree relative; ^2^Percentages do not total to 100 due to missing values; ^3^P-value is for comparison between NSAID users *versus* non-users in cases and controls.

Of the 1,034 subjects, 612 (59.2%) reported a history of NSAID use. Among these men, ASA use was more common than ibuprofen use (88.2% *vs.* 24.2%); some patients used both ASA and ibuprofen (12.4%). The association between NSAID use and the risk of advanced/aggressive prostate cancer is shown in Table 3. As noted previous, a decreased risk of disease was seen in both NSAID users (OR 0.67, 95% CI 0.52-0.86) and ASA users (OR 0.66, 95% CI 0.51-0.86), relative to NSAID non-users [15]. This decrease was also seen across increasing quintiles of intake. There was a trend towards a decreased risk of prostate cancer in ibuprofen users, although this did not reach statistical significance (OR 0.83, 95% CI, 0.56-1.21). This lack of significance may be due to the relatively small number of ibuprofen users in the study.

**Table 3:** Odds ratios and 95% confidence intervals for aggressive prostate cancer by use of NSAID medications.

**Medication**	**Exposed Cases (n)**	**Exposed Controls (n)**	**OR^1^**	**95% CI^1^**	**P-value**
NSAID^2^	272	340	0.67	0.52-0.86	0.002
Aspirin^3^	238	302	0.66	0.51-0.86	0.002
Ibuprofen^4^	73	75	0.83	0.56-1.21	0.310

^1^ OR odds ratios adjusted for age, race, and institution using logistic regression; CI confidence interval. ^2^ Referent category cases = 227, controls = 195.^3^ Referent category cases = 227, controls = 195. ^4^ Referent category cases = 227, controls = 195.

The dose and duration of NSAID use was also found to impact the risk of advanced/aggressive prostate cancer. Table 4 shows the risk of disease in NSAID and ASA users divided into quintiles based on extent of NSAID use. A similar analysis of ibuprofen use was not possible due to the small number of ibuprofen users. In both NSAID and ASA users, a stepwise decrease in the risk of prostate cancer was seen with increasing dose and duration of medication use. Compared to the first quintile, a significantly decreased risk of disease was seen in subjects in the fifth for both NSAID (OR 0.58, 95% CI 0.39–0.85) and ASA (OR 0.60, 95% CI 0.40–0.90).

**Table 4:** Odds ratios and 95% Confidence Intervals for aggressive prostate cancer by pills per year of NSAID use.

**Medication (pills per year)**	**Quintiles**					*P-*Trend
	1	2	3	4	5	
NSAID						
Median	0.2	1.0	3.0	6.0	15.5	
Cases (N)	57	58	62	35	55	
Controls (N)	65	61	63	68	83	
OR (95% CI)^1^	1.00	0.85 (0.57–1.27)	0.88 (0.60–1.30)	0.46 (0.29–0.71)	0.58 (0.39–0.85)	<0.01
Aspirin						
Median	0.3	1.0	3.0	5.3	15.0	
Cases (N)	48	54	47	32	52	
Controls (N)	57	55	56	58	76	
OR (95% CI)^1^	1.00	0.88 (0.58–1.33)	0.75 (0.49–1.15)	0.49 (0.31–0.78)	0.60 (0.40–0.90)	<0.01

^1^OR odds ratios adjusted for age, race, and institution using logistic regression; CI confidence interval.

We have previously reported an inverse association between NSAID use and risk of advanced/aggressive prostate cancer [10,15]. A similar, protective effect of NSAIDs has been reported by others [11,12,13,14,16,17,18,19,20,21], but remains controversial [22,23,24,25,26,27,28,29,30,31]. However, the majority of these studies investigated the impact of NSAIDs on all prostate cancer diagnoses [11,12,14,19,22,23,24,27,28,30] and few studied the association with aggressive disease [13,17,18,25,26,29]. Of the studies specifically investigating advanced or aggressive disease, most reported a protective effect of NSAIDs. Norrish *et al*. identified a stronger inverse association in “advanced” cases, defined as extracapsular disease extension or Gleason score ≥ 7, relative to all prostate cancer cases [18]. Leitzmann *et al*. reported no association between NSAID use and all cancers, but a trend towards a decreased risk of metastatic disease was noted in ASA users [25]. Mahmud *et al*. found that regular NSAID use was inversely related to the risk of detection of poorly differentiated cancers or those with higher percentage of core involvement on prostate biopsy [17]. The current study, restricted only to men with advanced/aggressive disease, shows a strong protective effect of NSAIDs on disease risk. These findings are interesting, as they suggest NSAIDs may preferentially inhibit the development of high-grade tumors, or prevent progression to advanced stage disease. 

The most intriguing results of our genetic analysis are shown in Table 5. For each inflammatory gene polymorphism, we first present their main association with prostate cancer. Next, patients were stratified by genotype, and the risk of disease in NSAID users relative to non-users was calculated. Genetic variants in several inflammatory pathway genes appeared to modify the protective effect of NSAIDs on prostate cancer development. For several SNPs, NSAID use reduced the risk of prostate cancer only in carriers of a particular genotype, whereas no association was seen in carriers of alternative genotypes. For example, in carriers of the AC + AA *MSR1* rs10503574 genotype, NSAID use markedly decreased the risk of prostate cancer (OR 0.31, 95% CI 0.15–0.62), whereas NSAIDs did not appear to alter disease risk in carriers of the AA genotype (OR 0.79, 95% CI 0.60–1.04) (*p*-interaction < 0.01). For other SNPs, a protective effect of NSAIDs was seen regardless of genotype, however, the specific genetic variants modified the magnitude of this effect. The remainder of the SNPs did not appear to materially alter the impact of NSAIDs on disease risk (not shown; available upon request). 

These data suggest that NSAID use may not result in a uniform protective effect across all individuals. The variation of NSAID effect according to genotype may further explain the conflicting results of previously published studies investigating NSAIDs and prostate cancer risk. Depending on the genetic makeup of study participants, NSAIDs may have a largely protective effect among certain individuals, but have no influence on prostate cancer development in others. This could impact any future recommendations that regular NSAID be used for prostate cancer prevention, as one might need to know an individual’s genotype before making such a recommendation.

**Table 5:** Association Between Candidate SNPs and Aggressive Prostate Cancer, and Modification of NSAID Protective Effect by these SNPs.

						**SNP Association^2^**					**NSAID Association^2^**					
**SNP^1^**	**Gene**	**Chromosome**	**Genotype**	**Cases (n)**	**Controls (n)**	**OR**	**95% CI**	**p-value**			**OR**	**95% CI**	**p-value**	
rs689470	*COX-2*	1	GG	413	455	1.00	referent				0.76	0.57–1.00	0.05	
			AG	58	65	1.25	0.78–2.01	0.36			0.29	0.13–0.65	0.002	
			AA	26	14	2.82	1.31–6.07	0.008			0.42	0.11–1.66	0.22	
											*p*-interaction^3^ = 0.06				
			GG	273	280	1.00	referent				0.83	0.59–1.17	0.29	
rs5030728	*TLR4*	9	AG	192	204	0.95	0.73–1.24	0.72			0.57	0.37–0.87	0.01	
			AA	33	50	0.66	0.41–1.06	0.09			0.29	0.11–0.73	0.009	
											*p*-interaction = 0.07				
			AA	118	120	1.00	referent				0.51	0.30–0.86	0.01	
rs4803455	*TGF-β1*	19	AC	216	248	0.88	0.64–1.21	0.43			0.64	0.44–0.94	0.02	
			CC	108	144	0.76	0.53–1.09	0.13			0.95	0.56–1.61	0.85	
											*p*-interaction = 0.06				
			AA	388	388	1.00	referent				0.64	0.48–0.86	0.003	
rs919766	*IL-12β*	5	AC+CC	105	145	0.73	0.54–0.97	0.03			0.81	0.48–1.38	0.44	
											*p*-interaction = 0.46				
			GG	416	462	1.00	referent				0.75	0.57–0.98	0.04	
rs11986822	*MSR1*	8	AG+AA	29	50	0.64	0.40–1.04	0.07			0.15	0.05–0.44	0.0006	
											*p*-interaction = 0.01				
			AA	418	435	1.00	referent				0.79	0.60–1.04	0.10	
rs10503574	*MSR1*	8	AC+CC	72	97	0.77	0.54–1.09	0.14			0.31	0.15–0.62	0.0009	
											*p*-interaction<0.01				

^1^ rs689470, rs5030728, rs4803455, rs919766, rs10503574 are intronic while rs11986822 is in a 3' untranslated region. Moreover, rs689470 was previously associated with prostate cancer (see text), whereas the other five were tagSNPs. ^2^ OR, odds ratio for prostate cancer for SNPs alone, and then in NSAID users *versus* non-users, within each genotype group (*i.e.*, among men who have each particular genotype). ^3^ P-interaction values permuted to address multiple testing.

The focus of this paper is on the potential effect modification of our previously reported inverse association between NSAID use and aggressive prostate cancer by genes that were marginally associated with disease. A number of statistical comparisons were made and fifty-two out of 311 SNPs exhibited associations with aggressive prostate cancer; of these 52, six (12%) showed potential interactions with NSAID use on risk of prostate cancer (Table 5). The number of associations observed here are substantially more than that expected based on chance alone. Since our aim was to generate hypotheses we have presented the nominal p-values. Nevertheless, in light of the multiple comparisons undertaken here these require further confirmatory work for confirmation

While many have speculated that chronic inflammation plays a key role in prostate carcinogenesis [39], the molecular mechanisms through which this effect occurs remain unclear. The genetic enzymes reported here may act as the molecular targets for NSAIDs and their metabolites, and further investigation of these proteins may shed light on the mechanisms through which NSAIDs may impact prostate cancer.

A number of these genes are well characterized and have been previously linked to prostate carcinogenesis. Increased *COX-2* expression has been reported in prostate tumors [40,41,42,43,44], and may be associated with high-grade lesions [45] or prostate cancer progression [46]. Others have reported that *COX-2* expression is not consistently elevated in prostate cancer cells themselves, but instead is overexpressed only in inflammatory cells or atrophic epithelial cells within the prostate [34]. Nonetheless, genetic variation in *COX-2* has been shown to alter the risk of prostate cancer irrespective of NSAID use. *TLR4* is a receptor molecule that initiates pro-inflammatory signaling pathways upon recognition of pathogen associated molecular patterns. Prior studies have reported differential *TLR4* expression in high-grade tumors relative to normal prostate tissue [47], and sequence variants in several *TLR* genes have been linked to prostate cancer risk [48]. *TGFβ* is thought to have an anti-inflammatory function, and it appears that genetic variation of this enzyme alters prostate cancer risk [49]. *IL-12*, an activator of natural killer cells and T-cells, has shown anti-tumor effects in a number of different cancer models [50,51,52]. In mice, *IL-12* transduced bone marrow cells were shown to suppress the development of metastatic prostate cancer [53]. Mutations of *MSR-1*, a transmembrane protein expressed on macrophages and involved in innate immunity, are associated with both hereditary and sporadic prostate cancer risk [54].

Furthermore, studies of various disease processes have suggested that NSAIDs interact with several of these gene products on the molecular level. The inhibition of COX-2 by NSAIDs is well characterized, and many have hypothesized that this inhibition is responsible for the potential chemoprotective effects of NSAIDs on prostate cancer development [55,56]. The gastroenterology literature has reported that NSAID-induced bowel injury may be due to an NSAID-triggered inflammatory response mediated by a TLR4-dependent signaling pathway [57]. In models of esophageal cancer, it has been proposed that TGFβ may serve to induce COX-2 expression [58]. Finally, experimental data has shown that NSAIDs are capable of inhibiting IL-12 folding and secretion; [59] and NSAID treatment results in decreased IL-12 expression in cervical cancer tissue [60]. Similar molecular interactions in the prostate may serve as the mechanism through which NSAIDs alter prostate cancer risk. 

As *COX-2* does not appear to be overexpressed in human prostate cancer cells, the protective effects of NSAID may function through *COX-2* inhibition of endogenous inflammatory infiltrates, or even through a *COX-2* independent pathway [34,39]. Of the other five genes found to modify the effect of NSAIDs in the current study, all are expressed on monocytes, macrophages, or other inflammatory cells. Thus, the mechanism through which NSAIDs alter prostate cancer risk may be through interaction with inflammatory infiltrates within prostate tissues, and not interactions with prostate epithelial cells themselves [34]. 

A final consideration is that inter-individual differences in NSAID metabolism may account for the differential effects of NSAIDs on prostate cancer development. Genetic variation could potentially result in differential NSAID metabolism resulting in increased intra-prostatic penetration of NSAIDs and their metabolites in certain individuals relative to others. These hypotheses warrant further research to clarify the mechanism through which NSAIDs may alter prostate carcinogenesis.

A limitation of the current study is that controls might not have been fully representative of the cases' source population because they were selected from men who underwent standard annual medical examinations. In comparison with the cases' source population, such controls might have cared more about their health, been more highly educated, had a higher income, been more likely to have medical insurance, or had medical conditions that increased their likelihood of NSAID use. As a proxy for these potential case-control differences, we adjusted our results for education and income; doing so did not materially alter our findings (not shown). Of course, since these factors are only proxies, adjusting for them may not have fully accounted for the potential case-control differences, especially with regard to medical conditions among controls. A related concern is potential bias arising from the lack of information about NSAID use other than aspirin and ibuprofen, and ensuing misclassification. If cases and controls used such medications in a similar manner, this bias would be nondifferential among cases and controls leading to an underestimation of the effect estimates. We would also expect a bias toward the null if controls were more likely to use non-measured NSAIDs than cases. Finally, there is potential for recall bias regarding the measured NSAIDs. The lack of general knowledge about the potentially protective effects of NSAIDs on prostate cancer should have made any such bias nondifferential among cases and controls. Moreover, we did not ask participants to specify the exact pill dose, which may also have resulted in nondifferential misclassification bias, and most likely an underestimate of the effect estimates.

## 4. Conclusions

This study identified genetic variants in inflammatory genes that may modulate our previously reported inverse association between NSAIDs and aggressive prostate cancer. The differential effect of NSAIDs according to genotype may partially explain the conflicting results of prior studies investigating NSAIDs and prostate cancer risk. Several inflammatory enzymes identified in this study may be key players in the pathway through which NSAIDs alter the risk of prostate cancer. Further investigation of these proteins may identify potential targets for future prostate cancer prevention therapeutics. 
